# Complete genome assembly of *Candida auris* representative strains of three geographical clades

**DOI:** 10.1128/mra.00882-23

**Published:** 2024-09-04

**Authors:** Nirmal Singh Mahar, Surbhi Kohli, Biswambhar Biswas, Immaculata Xess, Anil Thakur, Ishaan Gupta

**Affiliations:** 1 Department of Biochemical Engineering and Biotechnology, Indian Institute of Technology, New Delhi, India; 2 Regional Center for Biotechnology, Faridabad, Haryana, India; 3 All India Institute of Medical Sciences, New Delhi, India; University of California Riverside, Riverside, California, USA

**Keywords:** nanopore, long read sequencing, genomes, *Candida auris*, genome assembly, mycology, hybrid assembly, phylogenetic analysis

## Abstract

The complete genome assembly of *Candida auris* strains B11103, B11221, and B11244 is reported in this manuscript. These strains represent the three geographical clades, namely, South Asian (Clade I), South African (Clade III), and South American (Clade IV).

## ANNOUNCEMENT

*Candida auris*, first identified in Japan in 2009 (1), is a multidrug-resistant fungus recently added to the WHO’s Fungal Priority Pathogen List due to its global nosocomial outbreaks (2, 3).

Here, we report the chromosome-level assemblies of three strains of *Candida auris*, B11103, B11221, and B11244, representing three distinct geographical clades: South Asian (Clade I), South African (Clade III), and South American clades (Clade IV), respectively. These chromosome-level assemblies will be valuable for performing genomic studies on new isolates from these geographical clades.

*Candida auris* strains were obtained from the American Type Culture Collection, cultured overnight in 10 mL YPD (Yeast extract Peptone Dextrose) and harvested (4,000 × *g*, 5 min). Genomic DNA was extracted from yeast cells following a modified version of a previously described method (4). DNA isolation included lysis using lysis buffer and glass beads, followed by phenol-chloroform extraction (5), ethanol precipitation, and RNAse treatment.

Starting with 400 ng DNA, the nanopore kit SQK-LSK114 (Oxford Nanopore Technologies, Oxford, United Kingdom) was used for library preparation without any additional shearing to maintain large fragments. The process included end repair, purification, barcoding, and adaptor ligation as per the manufacturer’s guidelines. The kit’s long fragment buffer was used for size selection, enriching DNA fragments over 3 kb. The final library was adjusted to a 12-µL volume and 10–20 Fmol concentration.

These strains were sequenced using Oxford Nanopore Technology (ONT) on a MinION Mk1B sequencer with R10.4.1 chemistry. Guppy version 6.5.7 with SUP accuracy model was utilized to basecall the signals and trim the adapter sequences. The short-read data (6) from NCBI SRA (7) was preprocessed using bbduk.sh version 37.62 (8). Nanopore reads were polished using the filtered short-read data using FMLRC version 1.0.0 (9). The polished reads were further trimmed by CANU version 2.2 (10) to retain the high-quality reads. Reads with more than 5 kb read length were assembled using FLYE version 2.9.2 (11). The *de novo* assemblies were polished using Medaka version 1.7.2 (12) (three rounds) and Pilon version 1.24 (13) (five rounds), resulting in eight contigs, seven chromosomes, and one mitochondrial genome. The assembly metrics were generated using QUAST version 5.0.12 (14).

Benchmarking Single-Copy Orthologs (BUSCO) version 5.3.0 (15) with saccharomycetes_odb10 database was used to assess the quality of the final assemblies. Details about the assemblies' statistics and BUSCO scores are given in Table 1.

**TABLE 1:** Assembly statistics and other information for *Candida auris* strains^*a*^

Strain	Total ONT reads	Mean read length (kb)	Number of contigs	N50 (bp)	Longest contig (bp)	BUSCO score (%) (single copy, duplicated)	Guanine-Cytosine content (%)	Genome size (bp)	Mean sequencing depth (short read^*b*^, long read)
B11103	103,592	5.914	8	2,306,867	4,179,689	98.8 (98.6, 0.2)	45.19	12,416,283	78.18, 43.34
B11221	87,022	5.285	8	2,354,394	4,298,089	98.9 (98.8, 0.1)	45.18	12,403,051	79.47, 31.69
B11244	151,749	3.322	8	2,326,985	4,200,565	98.9 (98.8, 0.1)	45.20	12,407,872	73.97, 35.38

^*a*^ ONT, Oxford Nanopore Technology. GC, Guanine-Cytosine.

^*b*^ Short-read data were obtained from the previously published study (6).

Out of all seven contigs assembled per strain, three contigs were found to have start telomere sequence (A{1,2}ACCCAGACACCACCTAAGA{1,2}), and six contigs had end telomere sequence (TTTCTTAGGTGGTGTCTGGGT{1,2}). The telomere sequences were identified from the assembly using TelFinder version 1.0.0 (16).

**Fig 1:**
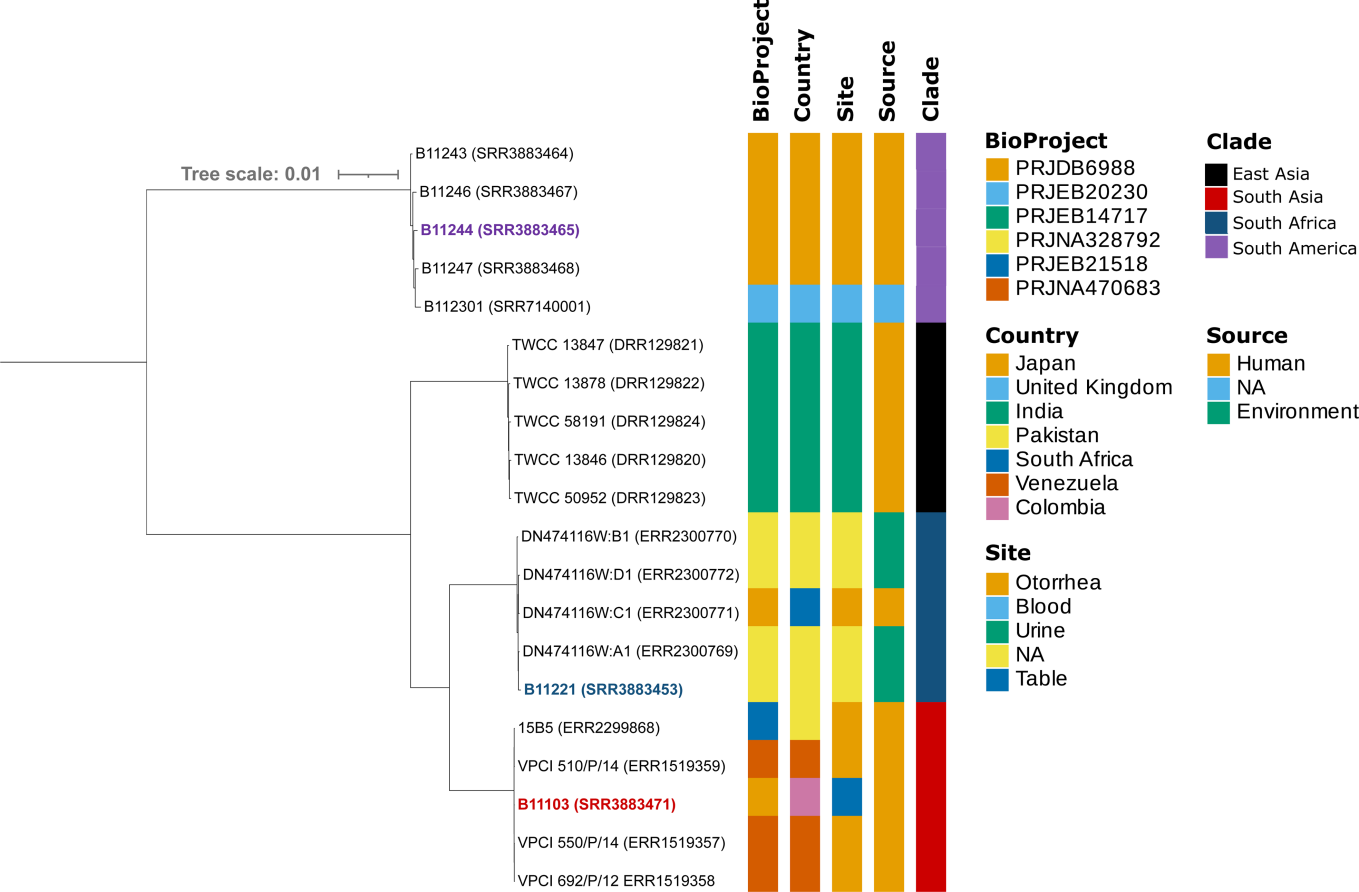
Phylogenetic tree showing four distinct geographical clades of *Candida auris*. The strains assembled in this study are highlighted in blue (B11221), red (B11103), and purple (B11244), which belong to South African, South Asian, and South American clades, respectively. The heatmap adjacent to the tree contains additional information about the strains utilized for the SNP analysis.

SNPs (single nucleotide polymorphisms) were called for the three strains using short-read sequencing data with GATK version 4.2.2.0 (17), and *Candida auris* B844 (18) (RefSeq accession GCA_002759435.2) was used as the reference genome. Publicly available data of strains from six projects [BioProject ID: PRJDB6988 (19), PRJEB21518 (not published), PRJEB20230 (20), PRJEB14717 (21), PRJNA328792 (6), and PRJNA470683 (22)] were also used for SNP calling (Fig. 1). A phylogenetic tree was then constructed from the SNP data using IQ-TREE version 2.2.2.7 (23) with a GTR + ASC model and visualized in the iTOL browser (24) (Fig. 1). Default parameters were used except stated otherwise.

## Data Availability

The whole-genome sequence and assemblies in this study are available in NCBI as follows: B11103, B11221, and B11244 under the BioProject PRJNA976115 and PRJNA976506, respectively. Raw long-read data for B11103, B11221, and B11244 are accessible via NCBI with the accession numbers SRR25919465, SRR25919458, and SRR25919459, respectively. Short-read data for B11103, B11221, and B11244 are accessible via NCBI with the accession numbers SRR3883471, SRR3883453, and SRR3883465, respectively.
